# Synthesis of Highly Thermally Stable Daidzein-Based Main-Chain-Type Benzoxazine Resins

**DOI:** 10.3390/polym11081341

**Published:** 2019-08-13

**Authors:** Mengchao Han, Sijia You, Yuting Wang, Kan Zhang, Shengfu Yang

**Affiliations:** 1 Research School of Polymeric Materials, School of Materials Science and Engineering, Jiangsu University, Zhenjiang 212013, China; 2 Department of Chemistry, University of Leicester, Leicester LE1 7RH, UK

**Keywords:** benzoxazine, daidzein, high thermal stability, polybenzoxazine

## Abstract

In recent years, main-chain-type benzoxazine resins have been extensively investigated due to their excellent comprehensive properties for many potential applications. In this work, two new types of main-chain benzoxazine polymers were synthesized from daidzein, aromatic/aliphatic diamine, and paraformaldehyde. Unlike the approaches used synthesizing traditional main-chain-type benzoxazine polymers, the precursors derived from daidzein can undergo a further cross-linking polymerization in addition to the ring-opening polymerization of the oxazine ring. The structures of the new polymers were then studied by ^1^H nuclear magnetic resonance spectroscopy (NMR) and Fourier-transform infrared spectroscopy (FT-IR), and the molecular weights were determined by using gel permeation chromatography (GPC). We also monitored the polymerization process by differential scanning calorimetry (DSC) and in situ FT-IR. In addition, the thermal stability and flame-retardant properties of the resulting polybenzoxazines were investigated using TGA and microscale combustion calorimeter (MCC). The polybenzoxazines obtained in this study exhibited a very high thermal stability and low flammability, with a *T_g_* value greater than 400 °C, and a heat release capacity (HRC) value lower than 30 J/(g K).

## 1. Introduction

1,3-Benzoxazine monomers are new types of thermosetting resins that have attracted wide attention over the past few decades due to the outstanding molecular design flexibility [1,2,3,4]. Benzoxazine monomers can be synthesized from primary amines, phenolic derivatives, and formaldehyde through Mannich condensation, and they can undergo thermally activated polymerization to form cross-linked polymeric networks, namely, polybenzoxazines, without adding catalysts/initiators. In addition to the unique advantage of benzoxazine monomers, polybenzoxazines also possess many excellent properties, such as near-zero shrinkage during polymerization [5], high thermal stability [6,7,8,9], low surface free energy [10,11], self-healing capacity [12], low dielectric constant [9,13,14], and low flammability [15,16,17]. These outstanding characteristics make benzoxazine monomers highly promising candidates for applications in high-performance areas, including electronic packaging, aerospace, coating, and adhesives [4].

However, the difficulty of thin-film processing and the brittleness of the cured polymers from the traditional benzoxazine monomers need to be overcome in order to extend the utilization of benzoxazine resins. The properties of thermosets cured from main-chain benzoxazines, in which benzoxazine groups form a repeating unit in the main chain, prove to be excellent compared with those derived from benzoxazine monomers [18,19]. In addition, benzoxazine resins have been facing the same challenges as most polymeric materials in terms of availability of starting materials from petrochemistry. Thus, developing new benzoxazine resins based on renewable resources has emerged as an efficient approach to realize the sustainable development of thermosets [20].

Daidzein (7-hydroxyl-3-(4-hydroxylphenyl)-4*H*-chromen-4-one), as a derivative found in soybean and other legumes [21], has been extensively used in the pharmaceutical and food industries due to its biological activities, such as antioxidation, antibacterial, and anti-inflammatory characteristics [22,23,24]. Moreover, daidzein is abundantly available all around the world since its weight concentration in soy germ is as high as 41.7%. Notably, each daidzein compound has two phenolic hydroxyls, making it an appropriate phenolic resource for synthesizing various thermosetting resins. Daidzein-based epoxy and benzoxazine resins have been recently synthesized, and the properties of the resulting thermosets have also been evaluated [25,26]. The daidzein-based epoxy resins exhibited excellent flame-retardant performance with a flammability rating of V-0 in the UL94 test [25]. In addition, a daidzein-based benzoxazine was also prepared, which showed very high thermal stability with a glass transition temperature as high as 391 °C [26]. 

In this work, we combine the advantages of the main-chain-type benzoxazine and the high performance of daidzein-based thermosets and synthesize two novel main-chain-type benzoxazines from daidzein, aromatic/aliphatic diamine, and paraformaldehyde. The detailed synthetic methods and the properties of polybenzoxazines derived from the new obtained main-chain-type benzoxazine resins are reported.

## 2. Experimental Section

### 2.1. Materials

1,6-Hexamethylenediamine (hda) and 4,4′-diaminodiphenylmethane (ddm) (98%) were purchased from Sigma-Aldrich. Daidzein, 1,4-dioxane and *N*,*N*-dimethylformamide (DMF) were obtained from East Instrument Chemical Glass Co., Ltd., Zhenjiang, China. All above chemicals were used as received with no purification.

### 2.2. Characterization

Nuclear magnetic resonance (NMR) spectra of benzoxazines in DMSO-*d_6_* were recorded using an NMR spectrometer (Bruker AVANCE II, 400 MHz, Bruker, Switzerland), and tetramethylsilane was used as the internal reference. A Nicolet Nexus 670 spectrophotometer (FT-IR, Nicolet, USA) was used to obtain the Fourier-transform infrared (FTIR) spectra. The molecular weight and dispersity index of the polybenzoxazine precursors were recorded by using a gel permeation chromatography (GPC) (GPC-20A, Shimadzu, Japan) at a flow rate of 1 mL/min with DMF as the solvent and polystyrene (PS) as the standard. Differential scanning calorimetry (DSC) was carried out using a NETZSCH DSC (NETZSCH, Germany) apparatus (model 204f1) with a heating rate of 10 °C/min in a nitrogen atmosphere. Dynamic mechanical analysis (DMA) was performed using a NETZSCH DMA/242E analyzer (NETZSCH, Germany) operated in the tension mode. An amplitude of 10 µm, a frequency of 1 Hz and a temperature ramp rate of 3 °C/min were applied during the DMA test. Thermogravimetric analysis (TGA) was carried out by using NETZSCH STA449-C apparatus (NETZSCH, Selb, Germany) in a temperature range from 25 to 850 °C. The measurement was in a heating rate of 10 °C/min under N_2_ or air atmosphere. The total heat release (THR, kJ/g) and the specific heat release rate (HRR, W/g) were obtained from a microscale combustion calorimeter (MCC) (Fire Testing Technology, United Kingdom) based on the ASTM 7309-2007a standard.

### 2.3. Synthesis of the Main-Chain-Type Benzoxazine Starting from Daidzein, 4,4′-Diaminodiphenylmethane and Paraformaldehyde (Abbreviated as Poly(Dd-Ddm)_Main_) 

The following compounds were mixed in the 250 mL round flask, which was equipped with a mechanical stirrer, a thermometer, and a reflux condenser: 40 mL of 1,4-dioxane, 10 mL of DMF, daidzein (0.63 g, 2.50 mmol), paraformaldehyde (0.32 g, 10.60 mmol), and ddm (0.49 g, 2.50 mmol). The mixture was stirred at 120 °C for 48 h. Afterward the mixture was cooled to room temperature and precipitated into 200 mL of deionized water. The precipitate was then filtered and dried at 60 °C in a vacuum oven. Finally, the solid product with yellow color was obtained (yield ca. 75%).

### 2.4. Synthesis of the Main-Chain-Type Benzoxazine Starting from Daidzein, 1,6-Hexamethylenediamine and Paraformaldehyde (Abbreviated as Poly(Dd-Hda)_Main_) 

A 250 mL round-bottom flask was added with 40 mL of 1,4-dioxane, 10 mL of DMF, daidzein (0.75 g, 3.00 mmol), paraformaldehyde (0.38 g, 12.70 mmol), and hda (0.34 g, 3.00 mmol). The mixture was stirred at 120 °C for 48 h. The mixture solution was then precipitated into 200 mL deionized water. The precipitate was then filtered and dried at 60 °C in a vacuum oven. Finally, the solid product with light yellow color was obtained (yield ca. 72%).

### 2.5. Polymerization of Daidzein-Based Main-Chain-Type Benzoxazines

The following method was used to prepare polybenzoxazine samples. The main-chain-type benzoxazines were dissolved in DMF to form 30% solid content solutions. Afterward, the solutions were transferred into a stainless steel mould. In order to remove the solvent in the samples, the samples were treated in an air-circulating oven at 120 °C for two days. Both main-chain types of benzoxazines, poly(Dd-ddm)_main_ and poly(Dd-hda)_main_, were then polymerized at 160, 180, 200, 220, 240, and 260 °C for 2 h each to obtain cross-linked poly(Dd-ddm)^X^_main_ and poly(Dd-hda)^X^_main_, respectively. 

## 3. Result and Discussion

### 3.1. Synthesis of Daidzein-Based Main-Chain-Type Benzoxazines

**Scheme 1:**
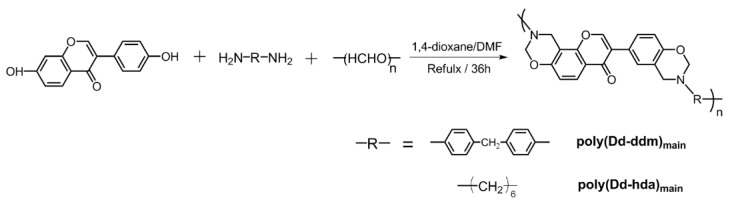
Synthesis of daidzein-based main-chain-type benzoxazines.

New main-chain-type benzoxazines derived from renewable resources have been successfully synthesized using diamines (DDM), paraformaldehyde, and daidzein as shown in Scheme 1. In general, the one-pot synthesis of main-chain type benzoxazines via Mannich condensation involves various intermediates, inculuding polytriazine, which is formed from the condensation between the diamines and formaldehyde [3]. We noticed that single component solvents such as toluene, xylenes, 1,4-doxane, and chloroform cannot achieve main-chain benzoxazines with satisfactory yields although these solvents are recommended as ideal solvents for synthesizing benzoxazine monomers. Both the starting materials, daidzein and the intermediate polytriazine, have poor solubility in these single component solvents. However, reacting in a mixture solvent, 1,4-doxane/DMF, which shows higher polarity, led to a homogeneous reaction solution with high yields (>70%). 

**Figure 1:**
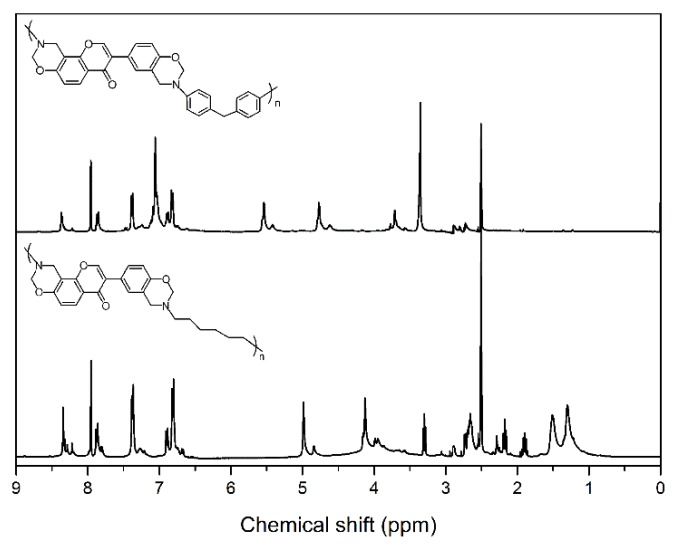
^1^H NMR spectra of poly(Dd-ddm)_main_ (**top**) and poly(Dd-hda)_main_ (**bottom**).

NMR spectroscopy was used to characterize the chemical structures of main-chain-type benzoxazines obtained in this study (see Figure 1 for the ^1^H NMR spectra of benzoxazines). The characteristic signals attributed to the benzoxazine structure, Ar–C*H*_2_–N– and –O–C*H*_2_–N– for poly(Dd-ddm)_main_ and poly(Dd-hda)_main_ were found at around 4.76 and 5.54 ppm and 4.13 and 4.99 ppm, respectively. Furthermore, the ^1^H NMR spectra confirmed the presence of a methylene group corresponding to the C*H*_2_ between the benzene rings in ddm for poly(Dd-ddm)_main_ at 3.71. In addition, the typical resonances for the protons in double bond =C*H*_2_ from daidzein for poly(Dd-ddm)_main_ and poly(Dd-hda)_main_ were observed at 7.95 ppm and 7.97 ppm, respectively. Moreover, the degree of ring closure was around 72% and 77% for poly(Dd-ddm)_main_ and poly(Dd-hda)_main_, respectively, as determined from the integral ratio of the resonances for the ring-opened methylene units and the oxazine methylene units. The ring-opened structures in the main-chain-type benzoxazines could not contribute to the ring-opening polymerization, but the phenolic groups in these structures could promote the polymerization process through the catalytic effect. 

The apparent molecular weights of poly(Dd-ddm)_main_ and poly(Dd-hda)_main_ were obtained by using GPC and the results are summarized in Table 1. The number-averaged molecular weights (Mn) of poly(Dd-ddm)_main_ and poly(Dd-hda)_main_ were estimated as 7019 and 5754 g/mol, while the dispersity index were determined as 1.39 and 1.44, respectively. The molecular weights of these main-chain-type benzoxazines are moderate, presumably due to the limited mobility of the polymer chains. 

### 3.2. Thermally Activated Polymerization Behaviors

**Figure 2:**
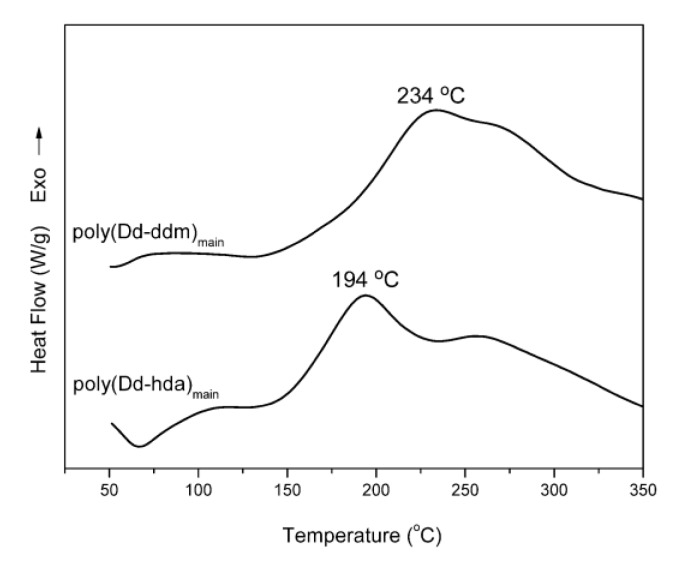
Differential scanning calorimetry (DSC) thermograms of poly(Dd-ddm)_main_ and poly(Dd-hda)_main_.

Nonisothermal DSC was carried out to study the polymerization of daidzein-based main-chain-type benzoxazines as shown in Figure 2. The DSC thermograms show that the maxima of the exothermic peaks of poly(Dd-ddm)_main_ and poly(Dd-hda)_main_ centered at 234 and 194 °C, respectively, which can be attributed to the ring-opening polymerization of oxazine rings [27]. Besides, both daidzein-based benzoxazines show an additional exothermic peak at around 260 °C, including another exothermic reaction occurring after the ring-opening polymerization of oxazine rings. These additional exothermic peaks partially overlapped with the exotherm of ring-opening polymerization of oxazine ring, which can be interpreted by the polymerization of the carbon–carbon double bond in the daidzein structure [26].

**Figure 3:**
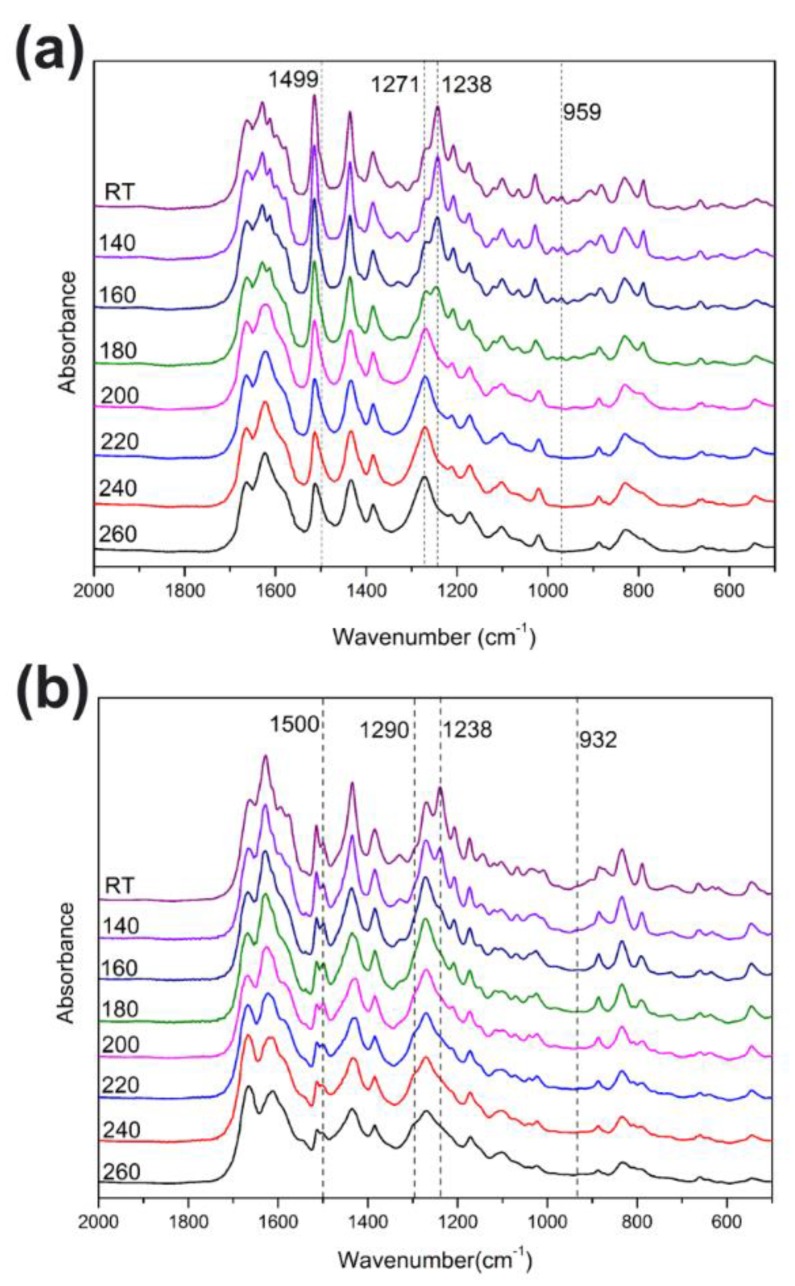
FT-IR spectra of poly(Dd-ddm)_main_ (**a**) and poly(Dd-hda)_main_ (**b**) obtained after various thermal treatments.

**Scheme 2:**
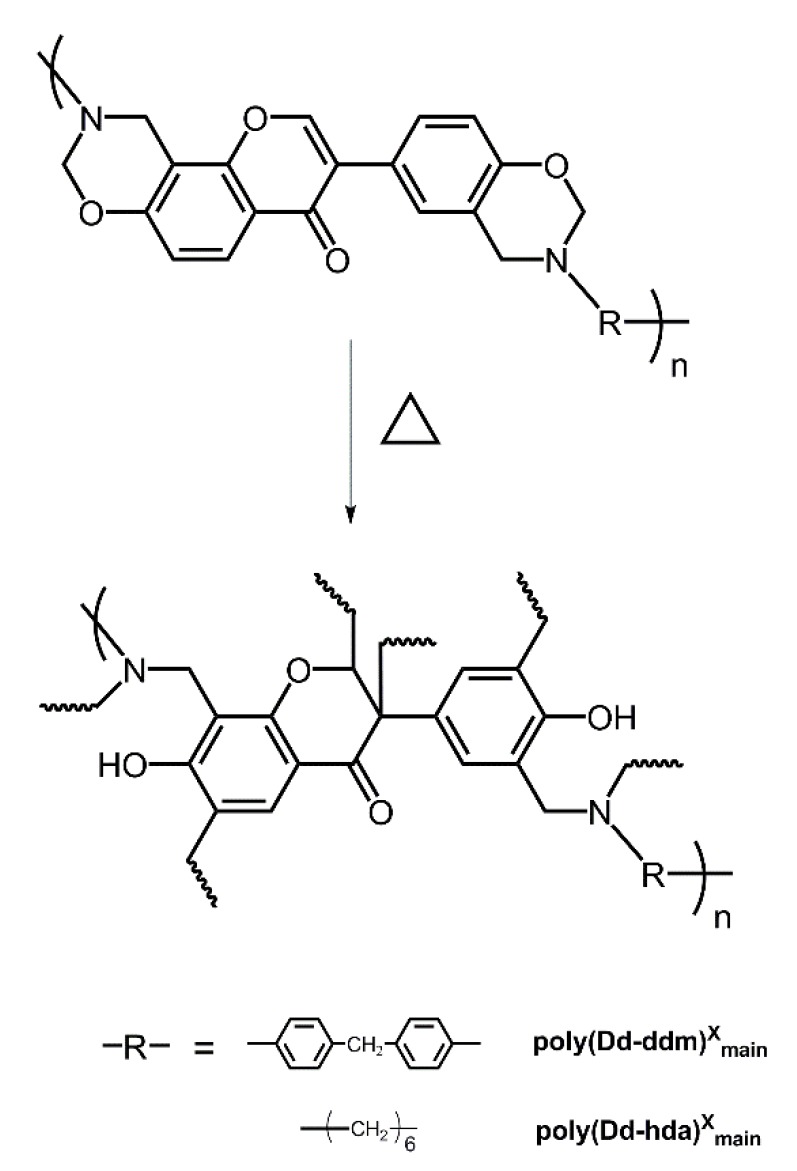
Possible polymerization behaviors of daidzein-based main-chain-type benzoxazines.

The polymerization behaviors of benzoxazines were also investigated using in situ FT-IR analyses. As shown in Figure 3, the characteristic bands at 1238 cm^−1^, which are due to the asymmetric stretching modes of C–O–C of poly(Dd-ddm)_main_ and poly(Dd-hda)_main_ gradually disappeared as the heating temperature increased [28]. The same tendency was found for bands at around 959 and 932 cm^−1^ for poly(Dd-ddm)_main_ and poly(Dd-hda)_main_, respectively, which can be attributed to the characteristic oxazine-related modes [29]. However, the intensity of both bands looks weaker than that of other reported benzoxazine monomers, which is caused by the existence of ring-opening structures in the main-chain backbone. These observations clearly indicate the ring-opening polymerization of the oxazine ring. Meanwhile, the characteristic olefin bands at 1499 and 1500 cm^−1^ decreased while the bands at 1271 and 1290 cm^−1^ increased for poly(Dd-ddm)_main_ and poly(Dd-hda)_main_, respectively, suggesting the further polymerization of the carbon–carbon double bond in the daidzein structure of both benzoxazines [25]. Moreover, no obvious changes were found for the band at 1270 cm^−1^ of poly(Dd-hda)_main_. The band at 1270 cm^−1^ can be assigned to the carbon–carbon stretching from the aliphatic segment in hexamethylenediamine, which was not involved in the curing process. The thermally activated polymerization processes of poly(Dd-ddm)_main_ and poly(Dd-hda)_main_ are described in Scheme 2. 

### 3.3. Thermal and Heat Release Properties of Polybenzoxazines

**Figure 4:**
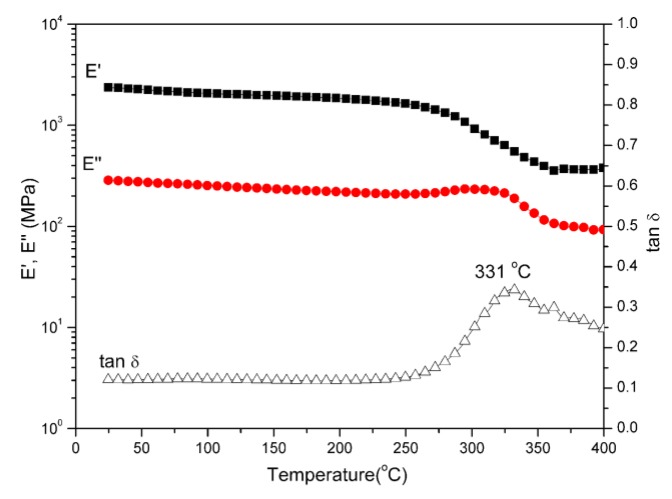
Dynamic mechanical spectra of poly(Dd-hda)^X^_main_.

**Figure 5:**
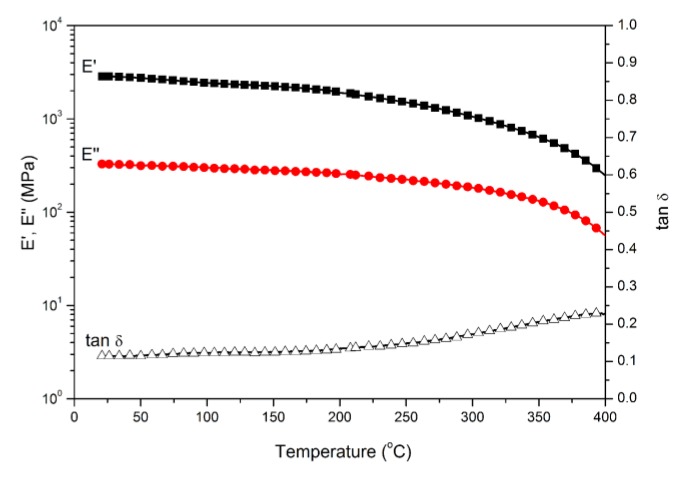
Dynamic mechanical spectra of poly(Dd-ddm)^X^_main_.

Polybenzoxazine thermosets were obtained after the thermally activated polymerization of main-chain-type benzoxazines. We performed DMA to study the thermomechanical properties of both polybenzoxazines derived from daidzein. Figure 4 and Figure 5 present the DMA profiles of poly(Dd-hda)^X^_main_ and poly(Dd-ddm)^X^_main_, respectively. As shown in Figure 4, the value of *T*_g_ for poly(Dd-hda)^X^_main_, determined as the peak temperature from the tan δ curve, was as high as 331 °C. However, poly(Dd-ddm)^X^_main_ exhibited no *T*_g_ before 400 °C, as seen in Figure 5, indicating that this polymeric material could be applied at a very high temperature. The values of *T*_g_ of both polybenzoxazines were much higher than those for other reported thermosets derived from main-chain-type benzoxazines [30,31,32]. In addition, the initial values of storage modulus (*E*’) and modulus loss (*E*’’) at 25 °C of poly(Dd-hda)^X^_main_ and poly(Dd-ddm)^X^_main_ were found as 2360 and 283 MPa and 2858 and 326 MPa, respectively, suggesting the relatively higher rigidity of poly(Dd-ddm)^X^_main_.

**Figure 6:**
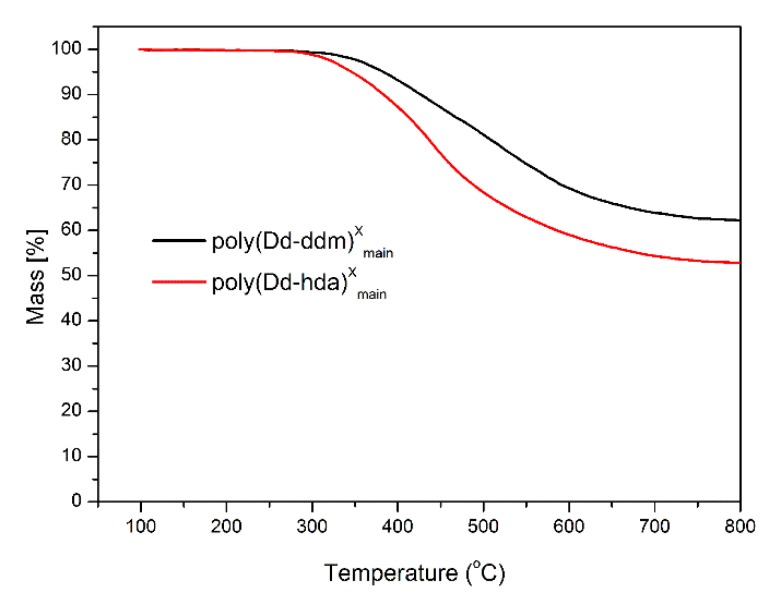
Thermogravimetric analysis of poly(Dd-hda)^X^_main_ and poly(Dd-ddm)^X^_main_ under N_2_.

**Figure 7:**
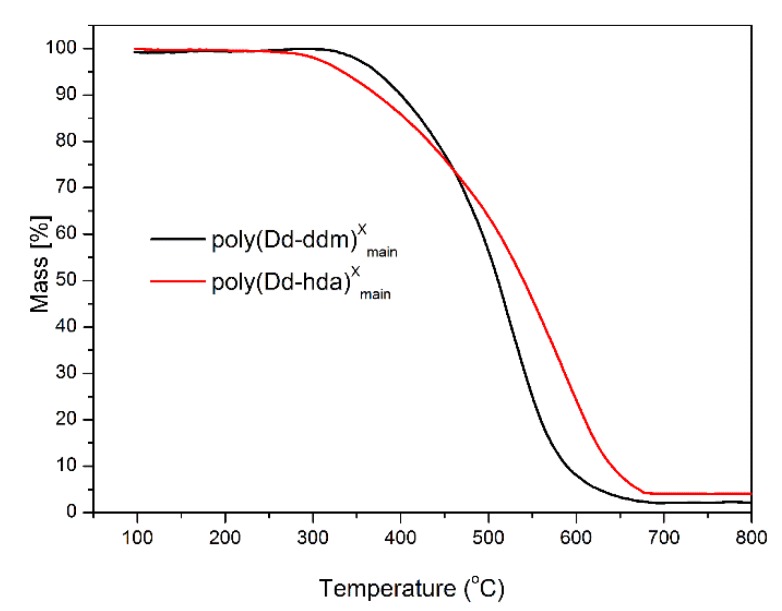
Thermogravimetric analysis of poly(Dd-hda)^X^_main_ and poly(Dd-ddm)^X^_main_ under air.

The thermal stability of poly(Dd-hda)^X^_main_ and poly(Dd-ddm)^X^_main_ was investigated by thermogravimetric analysis (TGA), and the corresponding weight-loss curves under nitrogen and air atmospheres are presented in Figure 6 and Figure 7, respectively. Poly(Dd-hda)^X^_main_ exhibits good thermal stability with the initial decomposition temperature of 5% weight losses (T_d5_) at 346 °C (in nitrogen) and 334 °C (in air), and the initial decomposition temperature of 10% weight losses (T_d10_) of 383 °C (in nitrogen) and 375 °C (in air). Additionally, poly(Dd-hda)^X^_main_ had a char yield value of 53% under N_2_ at 800 °C. Compared with poly(Dd-hda)^X^_main_, poly(Dd-ddm)^X^_main_ exhibited even-higher thermal stability, with T_d5_ values at 383 °C (N_2_) and 373 °C (air), and T_d10_ values at of 426 °C (N_2_) and 401 °C (air). The char yield value (Yc) for poly(Dd-ddm)^X^_main_ was obtained as high as 62% under N_2_, which is also much higher than the value for poly(Dd-hda)^X^_mai__n_. The higher thermal stability of poly(Dd-ddm)^X^_main_ can be attributed to the existence of highly aromatic cross-linked networks in polybenzoxazine. In addition, the char yield values obtained under air for both polymers were much lower than those obtained under N_2_. The ash generated in air condition could be the degraded products, such as carbides and oxycarbides derived from the degradation fragments. Moreover, the height of TGA curves was reversed due to the difference of the rates for the weight loss during TGA measurements. At the initial degradation stage, poly(Dd-ddm)^X^_main_ exhibited a lower weight-loss rate but higher thermal stability than poly(Dd-hda)^X^_main_. However, as the temperature increased to 450 °C, poly(Dd-ddm)^X^_main_ started to decompose quickly, and the degradation for poly(Dd-hda)^X^_main_ reached a steady trend, thus resulting in the reversed TGA curves. 

**Figure 8:**
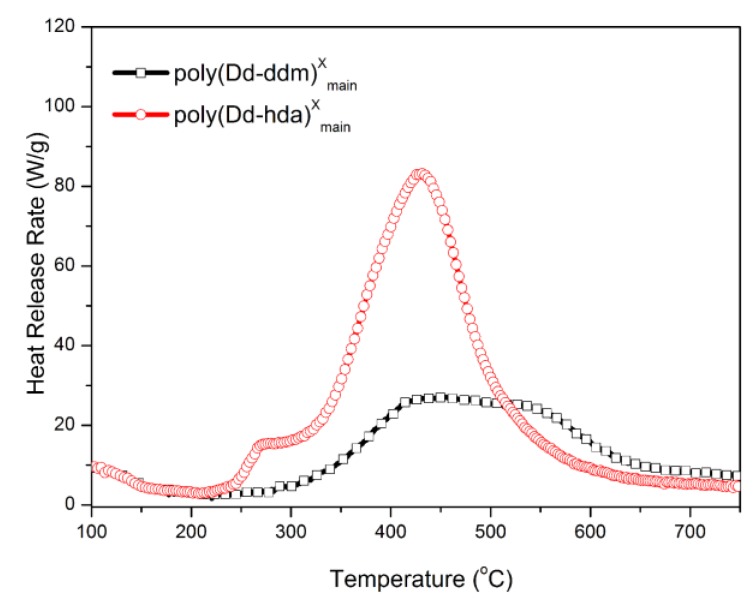
Heat release rate (HRR) versus temperature for poly(Dd-hda)^X^_main_ and poly(Dd-ddm)^X^_main_.

**Figure 9:**
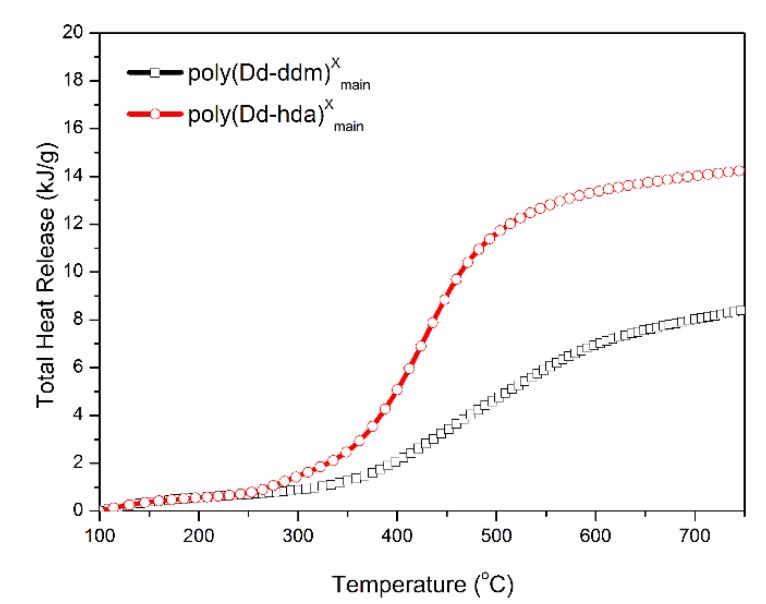
Total heat release (THR) versus temperature for poly(Dd-hda)^X^_main_ and poly(Dd-ddm)^X^_main_.

Finally, MCC was carried out to quantitatively evaluate the flammability of polybenzoxazines, including the heat release capacity (HRC) and total heat release (THR). HRC is recognized as an effective standard for evaluating the thermal combustion and a powerful predictor for the flammability of any given materials [33]. Figure 8 and Figure 9 show the graphics of HRR and THR as a function of the temperature, respectively. The MCC analysis allowed the calculation of the HRC values of poly(Dd-hda)^X^_main_ and poly(Dd-ddm)^X^_main_, which were found to be 83.2 and 27.1 J g^−1^K^−1^, respectively, while the corresponding THR values were calculated as 14.3 and 8.4 kJ g^−1^, respectively. Poly(Dd-ddm)^X^_main_ showed much lower flammability than poly(Dd-hda)^X^_main_, which is due to their structural difference. Poly(Dd-ddm)^X^_main_ was synthesized from the aromatic diamine while poly(Dd-hda)^X^_main_ was derived from the aliphatic diamine. In general, the aromatic polymers possess much higher thermal stability than aliphatic polymers since aromatic structures are easy to carbonize rather than simply decompose at the elevated temperature. Therefore, poly(Dd-hda)^X^_main_ yields highly carbonized structures during heating, resulting in both lower HRC and THR values. Notably, the HRC value of poly(Dd-ddm)^X^_main_ was much lower than those of polybenzoxazines obtained via polymerization of *ortho*-amide- or *ortho*-imide-functionalized benzoxazine resins [15,34]. Since materials with HRC values lower than 300 J g^−1^K^−1^ are generally regarded as self-extinguishing, whereas those with HRC below 100 J g^−1^K^−1^ are recognized as non-ignitable [35], both polybenzoxazines newly obtained in this study are non-ignitable polymeric materials. 

The data corresponding to the thermal stability and flame retardant properties of poly(Dd-hda)^X^_main_ and poly(Dd-ddm)^X^_main_ are summarized in Table 2, which suggests the excellent thermal stability and low flammability of both polybenzoxazines derived from daidzein-based main-chain-type benzoxazines. Our results therefore evidence the potential usage of both polybenzoxazines in fire resistant applications.

## 4. Conclusions

Two main-chain benzoxazine resins derived from daidzein were synthesized via one-pot Mannich condensation. The polybenzoxazine obtained from the herein synthesized partially bio-based main-chain-type benzoxazines exhibited excellent thermal stability, with *T*_g_ values greater than 400 °C. Furthermore, both thermosets obtained were found to be self-extinguishing and non-ignitable due to the low heat release capacity and the low total heat release. Our work demonstrates that the main-chain-type benzoxazines produced from natural renewable resources can be used as efficient polymeric matrix for producing high-performance fire-resistant materials.

## Figures and Tables

**Table 1:** Molecular weight of poly(Dd-ddm)_main_ and poly(Dd-hda)_main_.

Benzoxazine	Mn (Daltons)	Mw (Daltons)	Dispersity Index
poly(Dd-ddm)_main_	7019	9781	1.39
poly(Dd-hda)_main_	5754	8281	1.44

Mn—number-averaged molecular weight; Mw—weight-averaged molecular weight.

**Table 2:** Thermal and flame properties of polybenzoxazines.

Sample	*T*_g_ (°C)	N_2_			Air		HRC (J g^−1^K^−1^)	THR (kJ g^−1^)
		T_d5_ (°C)	T_d10_ (°C)	Yc	T_d5_ (°C)	T_d10_ (°C)		
poly(Dd-hda)^X^_main_	331	346	383	53	334	375	83.2	14.3
poly(Dd-ddm)^X^_main_	>400	383	426	62	373	401	27.1	8.4
